# Recent Advances of *Flowering Locus T* Gene in Higher Plants

**DOI:** 10.3390/ijms13033773

**Published:** 2012-03-21

**Authors:** Feng Xu, Xiaofeng Rong, Xiaohua Huang, Shuiyuan Cheng

**Affiliations:** 1 Hubei Key Laboratory of Economic Forest Germplasm Improvement and Resources Comprehensive Utilization, Huanggang Normal University, Huanggang, Hubei 438000, China; E-Mail: xufeng198@126.com; 2 College of Horticulture and Gardening, Yangtze University, Jingzhou, Hubei 434025, China; E-Mails: rongxf@sina.cn (X.R.); huangxiaohua87820@163.com (X.H.)

**Keywords:** flowering hormone, *FT* homologous genes, expression of products

## Abstract

*Flowering Locus T* (*FT*) can promote flowering in the plant photoperiod pathway and also facilitates vernalization flowering pathways and other ways to promote flowering. The expression of products of the *FT* gene is recognized as important parts of the flowering hormone and can induce flowering by long-distance transportation. In the present study, many *FT*-like genes were isolated, and the transgenic results show that *FT* gene can promote flowering in plants. This paper reviews the progress of the *FT* gene and its expression products to provide meaningful information for further studies of the functions of *FT* genes.

## 1. Introduction

Scientists have studied the flowering of higher plants for nearly a hundred years. The flowering process is extremely complex. Many studies have shown that the floral development process in plants is controlled not only by the complex regulation of multiple genes in internal factors, but also by a variety of environmental factors [1]. In recent years, the *Flowering Locus T* (*FT*) gene has been the most widely studied and effective gene in promoting early flowering in plants [2–4]. In addition, its homologous genes *PtFT1*, *CiFT*, *Hd3a*, and *SFT* have been isolated from poplar, citrus, rice, and tomato, respectively, and the overexpression of these genes can promote early flowering in transgenic plants [5–8]. The *FT* gene and its homologous genes are found in a variety of plants and therefore play similarly important roles; many studies have shown the protein encoded by the *FT* gene may be the major component of the “flowering hormone” [9,10]. In the present paper, some functions of the *FT* gene and research progress of the FT protein are briefly reviewed to further shed light on the function of *FT* genes and their products.

## 2. Flowering Hormone

The first researcher to study the flowering mechanism was Julis Sachs, who proposed the concept of the flowering material in 1865 following the flowering induction experiment [11]. He used a variety of related experiments to show that the floral promoting factor was first produced in leaves then transported over a long distance through the stem to produce buds that promote flowering. Chailakhyan first proposed the concept of the flowering hormone in 1936 and named this flowering-promoting material [12]. Experimental evidence confirmed that the flowering hormone as a common element in a variety of flowering plants. Such data have greatly stimulated research interest to identify the flowering hormone, but no result has been observed. Huang *et al.* [13] demonstrated the flowering hormone might be associated with the *FT* mRNA. This finding brought the attention of many scientists to the *FT* gene and its products. However, Corbesier *et al.* [14] subsequently suggested the *FT* mRNA is not a flowering hormone but an intermediate product, while the real flowering hormone should be the end product *FT* protein. This claim was proven by two experiments. First, FT protein was confirmed to promote flowering through long-distance transport, as observed using FT protein fused to green fluorescent protein (GFP). Second, grafting experiments of Arabidopsis mutants also proved that the FT protein could be transported over long distances and could ultimately promote flowering. In the course of the experiment, *FT* mRNA transport was not detected, indicating that the FT protein may be the major component of the flower hormone. Under a long-day photoperiod, the FT protein of Arabidopsis moves from leaves to the top and eventually interacts with Flowering Locus D (FD). The FT-FD complex formed directly or indirectly activates the flower-type genes, such as *APETALA1* (*AP1*) genes and ultimately promotes flowering in Arabidopsis [15,16]. The FT protein is part of the phloem transfer signal, which has been proven in the FT protein homologue in the phloem sap of rice, gourds, and Brassica [17–19]. Subsequently, a number of related studies proved that during the FT-mediated flowering process, the floral-promoting signal, which can be transferred from leaves to the apical meristem, is mainly composed of the FT protein [7,20–23].

## 3. Flower-Promoting Material *FT*

### 3.1. Flowering Pathway and Basic Functions of *FT* Genes

The *FT* gene was isolated as early as 1999, but only in more recent years have researchers been able to build a certain understanding about its regulatory networks and its homologous gene [9,24]. For example, in Arabidopsis, *FT* gene regulation first receives the signal from the photoperiod regulatory center gene *CONSTANS* (*CO*) under long-day conditions to modulate flowering. The *CO* transcription factor could interact with the promoter of FT gene [25]. Second, the integration of flower-induced signals by the *FT* gene from the photoperiod, autonomous, and vernalization pathways is promoted through the flowering integration gene *Suppressor of Overexpression Constans 1* (*SOC1*) and *Leafy* (LFY), which ultimately promote Arabidopsis flowering [26]. Therefore, the *FT* gene should be the final signaling target gene of the light cycle. The early flowering characteristic of transgenic plants with *CO* gene overexpression could be eliminated by *FT* gene mutations, whereas transgenic plants with *FT* gene overexpression exhibited early flowering in both short-day and long-day conditions [27]. Normally, *CO* and *FT* are expressed exclusively in vascular bundles, whereas *GIGANTEA* (*GI*) is expressed in various tissues. Interestingly, *GI* expressed in mesophyll or vascular tissues increases *FT* expression without up-regulating *CO* expression under short-day (SD) conditions. For example, ectopic expression test of *GI* demonstrated that *GI* could activate *FT* expression in a *CO*-independent manner under SD conditions through binding and weakening several *FT* repressors, such as SHORT VEGETATIVE PHASE (SVP), TEMPRANILLO (TEM)1, and TEM2 [28].

The *FT* gene can not only promote early flowering in plants, but also regulate developmental changes of seeds, pods, and other parts. Arabidopsis belongs to long-day plants. If suddenly placed in a short-day environment, the growth of plants may change minimally, but *FT* levels would be significantly reduced [16]. *FT* homologous genes in poplar control the development termination in short-day, dormancy initiation during vernalization, differentiation of buds, and other developmental processes [8]. In Norway Spruce, *FT* homologous genes regulate flower bud differentiation, formation of floral structures, and other transition processes, suggesting *FT* functions behave in a very conservative manner among the gymnosperms [29].

In addition to regulation by the photoperiod pathway, *FT* is also regulated by environmental temperature. At low temperature, low temperature signal in Arabidopsis is adjusted by the vernalization pathway and induces the development process by reducing the level of *Flowering Locus C* (*FLC*), which controls *FT* expression upstream [30–33]. On the other hand, temperature elevation leads to changes in the “ambient temperature transcriptome,” which are accompanied by an elevation in auxin levels, increased abundance/activity of Phytochromeinteracting Factor 4 (PIF4), and enhanced expression of *FT* [34]. Therefore, the photoperiod and temperature can independently regulate expression of *FT*.

### 3.2. *FT* Homologous Genes and Signal Transfer

The discovery of the *FT* gene function raises interest to study *FT* genes in different plants. The role of *FT* homologous genes has been extensively studied in heterologous and homologous plant systems. *FT* genes have been isolated from many plants, including crop plants, like *Oryza sativa* [5] and *Hordeum vulgare* [35]; fruit plants, like *Vitis vinifera* [36], *Malus domestica* [37], and *Pyrus communis* [38]; vegetable plants, like *Solanum lycopersicum* [7], *Cucurbita maxima* [19], *Solanum tuberosum* [39], *Pisum sativum* [40], and *Beta vulgaris* [41]; ornamental plants, like *Ipomoea nil* [42], *Chenopodium rubrum* [43], *Oncidium luridum* [44], and *Helianthus annuus* [45]. Overexpression of *FT* homologous genes in tomato, poplar, pear, and sunflower can promote early flowering, indicating the conserved function of *FT* homologous genes [7,46,47]. The *FT* gene belongs to a small gene family of floral regulators. In rice, 13 *FT*-like members was found. One of them, designated *RFT1*, lies adjacent to *Hd3a*, and its presumed amino acid sequence shows 91% identity with that of *Hd3a* [5]. Three members of *FT* were also found in barley [35]. Barley has an adaptive mechanism that adjusts flowering according to photoperiodic changes using a combination of different *FT*-like genes [48]. In addition, transcripts of *FT* homologous genes in grapes and maize have been shown to increase before flowering induction [33,49]. Xi and Yu [50] suggested that, in contrast to Arabidopsis and other plants, whose *FT* and other flowering genes are mainly expressed in the vascular tissues of leaves, *FT* homologous genes in most soybeans are highly expressed in the petiole during the flowering period. Thus, petioles may play an important role in the flowering regulation of soybeans and other plants, and may be the target organs of some flowering genes in soybean.

The *FT* signal can be transferred by grafting. Overexpression of the tomato *FT* homologous gene, *Single Flower Truss* (*SFT*), in tomato and tobacco, promotes early flowering. When the transgenic plant with constitutively expressed *SFT* is used as a scion to graft onto the rootstock of the *sft* mutant, SFT protein can induce early flowering of *sft*. However, when the *sft* mutant is used as a scion to graft onto the wild-type rootstock, it could not promote flowering, indicating a different *SFT* expression level, resulting in different outcomes [21]. Lin *et al.* [19] used different hops as rootstock and scion to detect FT protein transfer and extract the FT protein. Zhang [46] used transgenic plants as rootstock and non-transgenic plants as scion to perform a preliminary study on the grafting transfer of the *FT* signal. The results showed that the split-connection grafting method could not lead to early flowering of the non-transgenic scion, but after grafting, the flowering rate by heat-shock induction in transgenic rootstock significantly decreased, compared with that before grafting, indicating the *FT* signal in rootstock partially transferred after grafting. These studies showed *FT* must be transported over a long distance to the top to ultimately promote flowering.

## 4. *FT* Expression Product

*FT* expression products include *FT* mRNA and FT protein. In 2005, *FT* mRNA was considered the flowering hormone or its important component. This concept was later disproved, because previous experiments could not be repeated, and in later grafting experiments, FT protein, instead of *FT* mRNA, was finally detected. FT protein is a class of the CETS family, with a molecular weight of approximately 20 kDa [51]. This protein has a similar sequence and protein-folding structure to mammalian Raf Kinase Inhibitor Protein, and is homologous to the Phosphatidylethanolamine Binding Protein protein [52].

Lin *et al.* in 2007 [19] used Zucchini yellow mosaic virus-mediated transient expression of Arabidopsis in short-day pumpkin to illustrate the conservation of the FT protein [19]. The presence of the FT protein was also reported in rape [18]. The FT protein can be transported from the phloem to the top to induce flowering [19]. The rice *FT* homologue *Hd3a* was found in the inner region of the SAM and in stem and leaf blade vascular tissues, suggesting that it is produced in the vascular tissue of the leaf blade, transported through stem phloem tissue, unloaded at the upper end of the vascular tissue, and translocated to the SAM, probably through the region just beneath the SAM. These results suggest that the Hd3a protein, but not *Hd3a* mRNA, is a candidate for the florigen in rice [23]. Furthermore, Hd3a interacts with 14-3-3 proteins in the apical cells of shoots, yielding a complex that translocates to the nucleus and binds to the *Oryza sativa* FD1 transcription factor, a rice homologue of *Arabidopsis thaliana* FD. The resultant ternary “florigen activation complex” (FAC) induces transcription of *OsMADS15*, a homologue of *AtAPETALA1* (AP1), which leads to flowering. Taoka *et al*. in 2011 [53] have determined the 2.4 Å crystal structure of rice FAC, which provides a mechanistic basis for florigen function in flowering. The results indicated that 14-3-3 proteins act as intracellular receptors for florigen in shoot apical cells, and offer new approaches to manipulate flowering in various crops and trees. By these successful experiments, the FT protein is seen to have the features of most flowering hormones. It is conservative and can be transported over a long distance to the apical meristem. The FT protein can be detected in repetitions of the same experiment and experiments with different materials, but the FT protein cannot be concluded to be the flowering hormone. Liu *et al*. in 2008 [54] used a turnip mosaic virus with deletion of Coat Protein (CP) (TCVΔCP) as the vector, cloned *FT* and m*FT* (start codon was mutated into stop codon) sequences into the location of the original CP sequence transformed into Arabidopsis, and detected their sequences in unvaccinated upper new leaves and buds to investigate whether *FT* mRNA has a role in transferring important signals during plant growth and development. In contrast, the *GFP* sequence, which was added as a control variable, was not detected in the same area. These results showed that FT and m*FT* sequences are able to restore the intercellular mobility of TCVΔCP.

## 5. Prospects

Flower development is an important decision of higher plants and is regulated by a variety of flowering genes. The *FT* gene mainly affects the induction of flowering. Studies have identified its homologous genes in a variety of garden plants, fruit trees, and vegetables. Transgenic technology confirmed that the gene can promote early flowering in plants. Thus, the genetic engineering approach can be used to perform genetic reformation on the flowering genes in wood, such as ginkgo, to shorten their young period.

*FT* genes are conserved among different species. Although the *FT* gene and its expression products are somewhat understood, many issues still need to be further studied. One issue is the difference in expression and functions of *FT* genes between gymnosperms and angiosperms, between annual and perennial plants, and between different parts of the same plant, and how its expression is regulated. Although *FT* mRNA is not detected during the transfer process, whether it is unrelated to the flowering hormone is another issue to be considered. Moreover, it need to be ascertained whether the reasons that the FT protein has not been detected for a long time are its special structure or low content, the type of *FT* gene promoter, and how it is regulated. Likewise, more topics of concern are whether the present *FT* signal graft transfer is of high practical value, and what the transport mechanism and distribution pattern during *FT* grafting are. Answers to these questions are not clear now, and many related minor issues have also arisen. When these issues are satisfactorily resolved, the concept of the “flowering hormone” and the *FT* gene and its expression product will be better understood. The resolution will also be a guide to control the flowering of fruit trees, flowers, and vegetables.
